# *Rhus verniciflua* Stokes (RVS) and butein induce apoptosis of paclitaxel-resistant SKOV-3/PAX ovarian cancer cells through inhibition of AKT phosphorylation

**DOI:** 10.1186/s12906-016-1103-3

**Published:** 2016-04-27

**Authors:** Hyeong Sim Choi, Min Kyoung Kim, Youn Kyung Choi, Yong Cheol Shin, Sung-Gook Cho, Seong-Gyu Ko

**Affiliations:** Department of Science in Korean Medicine, Graduate School, Kyung Hee University, 1 Hoegi, Seoul, 130-701 Korea; Jeju International Marine Science Center for Research and Education, Korea Institute of Ocean Science & Technology (KIOST), Jeju, 695-975 Korea; Department of Preventive Medicine, College of Korean Medicine, Kyung Hee University, 1 Hoegi, Seoul, 130-701 Korea; Department of Biotechnology, Korea National University of Transportation, 61 Daehakro, Jeungpyeong-gun, Chungbuk, 368-701 Korea

**Keywords:** *Rhus verniciflua* Stokes, Butein, AKT, Paclitaxel-resistant ovarian cancer, Apoptosis

## Abstract

**Background:**

*Rhus verniciflua* Stokes (RVS) belongs to the Anacardiaceae family and traditionally used for cancer treatment. RVS and butein, a major compound of RVS, were known to induce apoptosis via AKT inhibition in cancer cells. Thus, in this study, we investigated the effect of RVS and its derivative compounds (fisetin, quercetin, butein) on cell death in SKOV-3/PAX cells.

**Methods:**

The 80 % ethanol extract of RVS and its derivative compounds (fisetin, quercetin, butein) were prepared. The cytotoxicity was measured by 3-(4,5-dimethylthiazol-2-yl)-2,5-diphenyltetrazolium bromide (MTT) colorimetric assay. Apoptotic cells were detected by staining with propidium iodide (PI) and Annexin V-fluorescein isothiocyanate/7-aminoactinomycin D (Annexin V-FITC/7-AAD). The expression level of intracellular signaling related-proteins in apoptosis and growth were measured by western blot assay.

**Results:**

We found that RVS and butein suppressed the growth of SKOV-3/PAX cells in a dose-dependent manner. We also found that RVS and butein produced the cleavage of caspase-9, -8, -3, and PARP. Similarly, sub-G_1_ phase and Annexin V-FITC positive cells were increased by RVS and butein. Moreover, RVS and butein significantly reduced AKT phosphorylation in SKOV-3/PAX cells. PI3K inhibitor LY294002 caused PARP cleavage supporting our finding.

**Conclusion:**

Our data clearly indicate that RVS and butein induce apoptosis of SKOV-3/PAX cells through inhibition of AKT activation. RVS and butein could be useful compounds for the treatment for paclitaxel resistant-ovarian cancer.

## Background

Cancer is one of disease and is the second leading cause of death all over the world [1, 2]. Although the incidence of ovarian cancer is the eighth in developed and developing countries in women. However, the mortality rate for ovarian cancer is the fifth and higher in more developed countries than less developed countries. Further reductions in cancer death rates can be improved by applying various therapies including surgery, radiation chemotherapy, hormonal therapy, and chemotherapy [3].

One of the most common chemotherapy agent used for the treatment of ovarian cancer patients are taxanes, such as paclitaxel [4, 5]. Despite many studies on therapy for decades, there are still considerable problems to be solved such as multi-drug resistant (MDR) [6, 7]. MDR plays a key role in chemotherapy because of existing anti-cancer drugs effectiveness wearing of as tumor build tolerance to them. Although ovarian cancer is sensitive to paclitaxel in early days, tumors ultimately are not in patients because of MDR [8, 9]. One of the acquisition mechanisms to paclitaxel is known as activation of the AKT [10, 11]. Phosphorylated AKT (p-AKT) is lead to an increase in cell growth but it inhibits cell death [12, 13]. Accordingly, the combination of AKT inhibitors with chemotherapy agent has been shown to sensitize ovarian cancer patients [14, 15].

*Rhus verniciflua* Stokes (RVS) has many useful effects such as it loosen the extravasated blood in the liver, control inflammation, and help the digestive organs in Donguibogam or a botanical list. Moreover, RVS is documented to be used for treating various diseases such as obesity, allergic inflammatory, allergic contact dermatitis, swelling, angiogenesis, Parkinson’s disease, Huntington’s disease, and cancer [16–18]. RVS is composed of diverse compounds such as butein, fisetin and quercetin [19].

Previous studies have demonstrated that RVS and butein induce cell death via AKT pathway in cancer cells [20, 21]. However, it is not reported that RVS or butein is lead to cell death through blocking AKT activation in paclitaxel-resistant ovarian cancer cells. In this study, we wanted to know that whether RVS of ethanol extracted can be induced apoptosis in paclitaxel resistant ovarian cancer. We have shown that RVS was inhibited AKT-mediated proliferation via inducing apoptosis. Moreover, butein, a compound of RVS, showed the same results as the RVS. Therefore, our results indicate that RVS and butein may be potential anticancer drug for paclitaxel resistant ovarian cancer.

## Methods

### Plant materials

*Toxicodendron vernicifluum* Stokes, formerly known as *Rhus verniciflua* Stokes (RVS) and the common name is Chinese lacquer tree. RVS belongs to the Anacardiaceae family and deciduous tree species [19, 22]. It is about 10 ~ 20 meter in height and divided into yellow endothelial and gray outer. The purpose of its cultivation is mainly used to paint with lacquer collected the sap or get wax from the fruit. Before you use it for food or medicine, it should be removed completely toxic due to the poisonous of RVS [23].

### Preparation of RVS extracts and chemical compounds

80 % ethanol extract of RVS used in this research was provided from Hanpoong pharm (Jeonju, Republic of Korea). The powder form of RVS was dissolved in 30 % ethanol to make a stock solution and stored at -80 °C until ready for use. Fisetin (PubChem CID: 5281614) and quercetin (PubChem CID: 5280343) were purchased from Sigma (St. Louis, MO). Butein (PubChem CID: 5281222) was obtained from Santa Cruz Biotechnology (Santa Cruz, CA, USA).

### Cell lines and cell culture

The paclitaxel resistant ovarian cancer cells, SKOV-3/PAX cells were provided by Prof. Cho. These cells were grown in Dulbecco’s modified Eagle’s medium (DMEM) supplemented with 10 % heat-inactivated fetal bovine serum (FBS), 1 % antibiotic-antimycotic solution, and 5nM of paclitaxel in a humidified atmosphere of 5 % CO_2_ at 37 °C.

### Cell proliferation assay

For cell proliferation assay, SKOV-3 or SKOV-3/PAX were seeded at 5x10^3^ cells in 96 well plates and then treated with paclitaxel (0, 1,10, 100 and 1000nM), RVS (0, 50, 100, 200 and 500 μg/mL), or butein (0, 2.5, 5 and 10 μg/mL) for 72 hours. Thereafter, cell viability was performed by 3-(4,5-dimethylthiazol-2-yl)-2,5-diphenyltetrazolium bromide (MTT) colorimetric assay with an absorbance at 570 nm.

### FACS analysis

Dead cells were measured by propidium iodide (PI) staining because PI is bound to DNA and can be used to detect abnormal cell populations. SKOV-3/PAX cells were treated with RVS and butein for 24 hours, and then fixed 70 % ethanol in ice-cold PBS at -20 °C. Next day, the fixed cells were resuspended in PBS containing 50 μg/mL of PI and 50 μg/mL of RNAse A, and incubated in the dark for 15 minutes at room temperature. The DNA contents of samples were analyzed by FACSCalibur flow cytometer (Becton-Dickinson, San Jose, CA). Apoptotic dead cells were detected by Annexin V-fluorescein isothiocyanate (FITC) and 7-aminoactinomycin D (7-AAD) staining. Annexin V-FITC, staining for phosphatidylserine (PS) in cell membrane, is detected in early apoptosis. 7-AAD is a membrane impermeability dye that is generally stained apoptotic cells and excluded viable cells. SKOV-3/PAX cells were treated with RVS and butein, and then harvested in Annexin V binding buffer. The samples were stained with Annexin V-FITC in the dark for 15 minutes at room temperature and gave more staining with 7-AAD in the same way. 2-dimensional staining to measure apoptosis and necrosis was subjected to FACSCalibur.

### Western blot

Whole lysates, proteins extracted from SKOV-3/PAX were separated by 10-15 % SDS-PAGE according to their molecular weights. Each protein was transferred to nitrocellulose blotting membrane and blotted by appropriate antibodies for following molecules at 4 °C; Anti-cleaved caspase-9 (sc22182), -p-p38 (sc7973), -p-JNK (sc6254), -ERK (sc1647), and -p-ERK (sc7383) antibodies were purchased from Santa Cruz Biotechnology (Santa Cruz, CA, USA). Anti-cleaved caspase-8 (#9496), -cleaved caspase-3 (#9661), -PARP (#9542), -p-Raf-1 (#9421), -AKT (#9272), -p-AKT (S473) (#9271), -JNK (#3708), and -p38 (#9212) antibodies were obtained from Cell Signaling Technology (Danver, MA, USA). Anti-α-tubulin (T5168) antibody was purchased from Sigma (St. Louis, MO). Next day, the membranes were washed three times in phosphate buffered saline with 0.01 % tween-20 (PBST) and incubated at room temperature for 1 hour with secondary antibodies. The membranes were then washed with PBST three times and visualized the protein bands using an enhanced chemiluminescence detection system (DoGen-Bio, Seoul, Korea) and exposed to X-ray film (Agfa-Gevaert N.V., Mortsel, Belgium).

### Statistical analysis

All experimental data were shown as mean ± SD and analyzed by Student *t*-test using Microsoft excel software. *P* value less than 0.05 was considered statistically significant.

## Results

### RVS inhibits cell proliferation in chemoresistant SKOV-3 cells

**Fig. 1:**
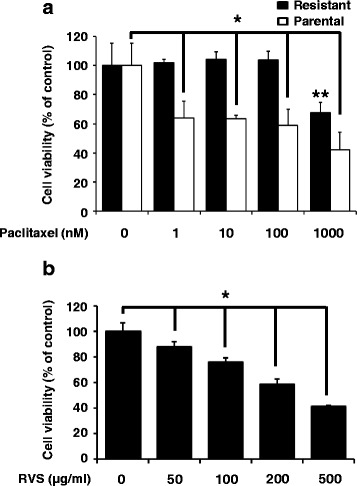
Proliferation of parental and paclitaxel resistant ovarian cancer cells by paclitaxel or RVS treatment for 72 hours. **a** The effect of paclitaxel on the viability in SKOV-3 or SKOV-3/PAX cells was determined by MTT assay. **b** The effect of RVS on the viability in SKOV-3/PAX cells was determined by MTT assay (mean ± SD; *n* = 6). *, *p* < 0.05 versus control in parental. **, *p* < 0.05 versus control in resistant

We first examined that whether SKOV-3/PAX cells were acquired resistant to paclitaxel from parental cells. As shown in Fig. 1a, paclitaxel inhibited growth of SKOV-3 cells, but not in SKOV-3/PAX cells. Next, we treated with RVS to identify the anti-proliferation effects in paclitaxel-resistant cells. As shown in Fig. 1b, RVS suppressed in a-dose dependently cell growth in SKOV-3/PAX cells. Therefore, our results indicate that RVS effectively inhibits the proliferation of paclitaxel-resistant ovarian cancer cells.

### RVS induces apoptosis in paclitaxel-resistant ovarian cancer cells

**Fig. 2:**
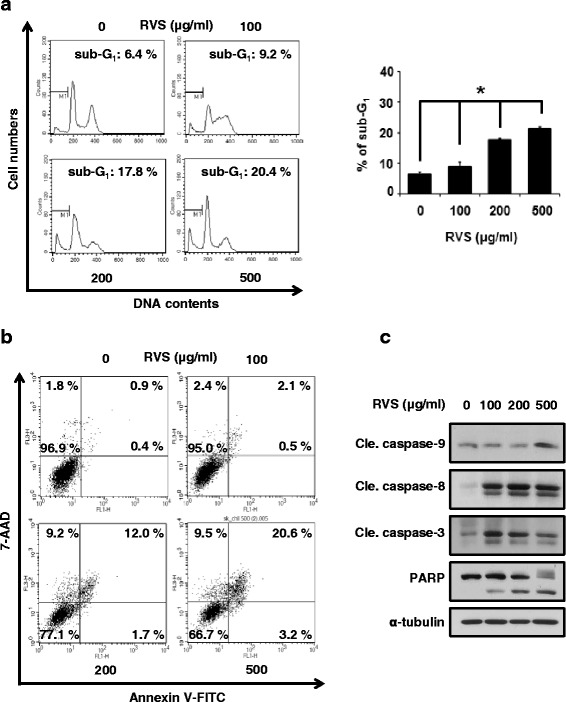
RVS induces apoptotic-cell death in paclitaxel resistant ovarian cancer cells for 24 hours. **a** Left panel, the cytotoxic effect of RVS in SKOV-3/PAX cells was determined by PI staining assay. Right panel, data represent quantitative results for left panel (mean ± SD; *n* = 3). *, *p* < 0.05. **b** The dead cells were differentiated between apoptosis and necrosis by Annexin V-FITC and 7-AAD dual staining. **c** The expression of procaspase-9, cleaved caspase-9, cleaved caspase-8, procaspase-3, caspase-3, and PARP in cell lysates from SKOV-3/PAX cells treated with RVS was determined by Western blot assay

To determine whether RVS could induce the cell death in SKOV-3/PAX cells, we were treated at various concentrations of RVS. First, we were stained DNA by PI and detected dead cells. The sub-G_1_ phases, dead cells, were increased by RVS in a dose-dependently manner. Especially, dead cell population showed that 500 μg/ml of RVS induced the sub-G_1_ phase to 20.4 % compared with control (Fig. 2a). Next, in order to whether RVS induces cell death inducing apoptosis or necrosis, we performed Annexin V-FITC and 7-AAD staining. SKOV-3/PAX cells were stained with Annexin V-FITC and 7-AAD, and then measured the proportion of apoptosis and necrosis-related cell death (Fig. 2b). As a result, it was increased the cell death due to apoptosis more than necrosis. Lastly, we were observed the change of apoptosis-related proteins. Cleaved form of PARP, caspase-9, -8, and -3 were increased by RVS treatment in SKOV-3/PAX cells (Fig. 2c). Therefore, our data show that RVS induces apoptosis through the caspase-dependent pathway.

### Butein suppresses cell proliferation and enhances apoptosis in paclitaxel-resistant ovarian cancer cells

**Fig. 3:**
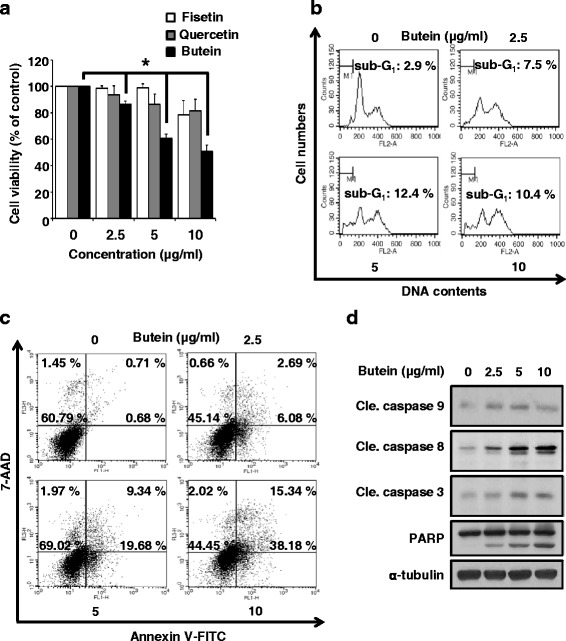
The effect of apoptosis in butein-treated SKOV-3/PAX cells. (**a**) The effect of butein, fisetin, and quercetin on the viability in SKOV-3/PAX cells for 72 hours was determined by MTT assay. (**b**) The cytotoxic effect of butein in SKOV-3/PAX cells for 24 hours was determined by PI staining assay. Bottom panel, data represent quantitative results for top panel (mean ± SD; *n* = 3). *, *p* < 0.05 versus control. (**c**) The dead cells were differentiated between apoptosis and necrosis by Annexin V- FITC and 7-AAD dual staining. (**d**) The expression of procaspase-9, cleaved caspased-9, cleaved caspase-8, procaspase-3, caspase-3, and PARP in cell lysates from SKOV-3/PAX cells treated with butein were determined by Western blot assay

We also wanted to know that whether which compounds of RVS was induced cell death in paclitaxel resistant ovarian cancer cells. As shown in Fig. 3a, butein significantly reduced cell growth than other compounds of RVS, such as fisetin or quercetin. It is reported that fisetin induces apoptosis in human non-small cell lung cancer via inhibiting MAPK signaling pathway and quercetin increases the apoptosis rate of ovarian cancer cells [24, 25]. To confirm whether butein caused apoptotic cell death, we treated with butein on SKOV-3/PAX cells. Like RVS, butein dose-dependently increased sub-G_1_ phase (Fig. 3b) and induced the apoptotic-cell death (Fig. 3c). Moreover, butein increased levels of activated caspase-9, -8, -3, and cleaved PARP in SKOV-3/PAX cells (Fig. 3d). Therefore, our data demonstrate that butein is the active compound of RVS and contributes to paclitaxel-resistant ovarian cancer cell apoptosis via caspase-dependent signaling.

### RVS and butein disrupt cell growth via AKT pathway in paclitaxel-resistant ovarian cancer cells

**Fig. 4:**
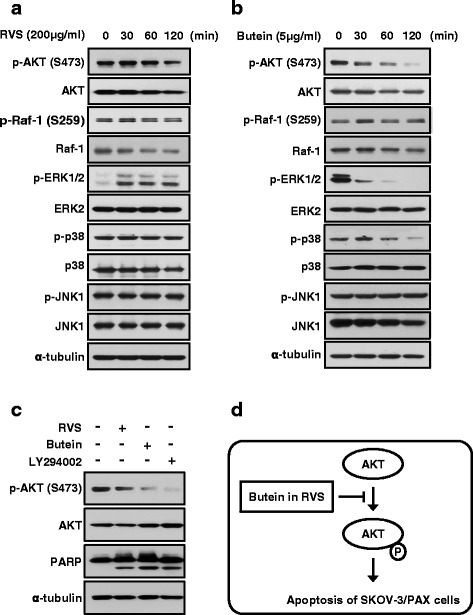
RVS and butein inhibit AKT signaling in SKOV-3/PAX cells for 2 hours. (**a** and **b**) The expression of AKT, Raf-1, ERK, p38, and JNK in cell lysates from SKOV-3/PAX cells treated with RVS or butein were determined by Western blot assay. (**c**) SKOV-3/PAX cells were pretreated with AKT inhibitor, LY294002 (50 μM) for 1 hour and then added with RVS or butein for another 24 hours. The expression levels of AKT phosphorylation and PARP cleavage were determined by Western blot assay. (**d**) A schematic model for the apoptosis via inhibition of AKT activity by RVS or butein

Further our understanding of the molecular effect of RVS or butein in growth control, we performed cells were treated with RVS or butein for 24 hours. When SKOV-3/PAX cells were treated with RVS, phosphorylation of AKT were reduced in a time-dependently manner (Fig. 4a). But phosphorylation of ERK was induces by RVS. In the case of butein, phosphorylation of ERK and p38 were decreased as well as AKT activity (Fig. 4b). Our data showed that inhibition of AKT activity was decreased by both RVS and butein, also the AKT signaling pathway is well known as a key role in cancer cell growth. Therefore, we showed that inhibition of AKT phosphorylation using by AKT inhibitor, LY294002, the PARP cleaved was increased by as much as RVS and butein (Fig. 4c). These results demonstrated that RVS and butein are inducing apoptosis through AKT signaling pathway (Fig. 4d).

## Discussion

Numerous reports are shown that AKT phosphorylation is induced in ovarian cancer patients and paclitaxel-treated in ovarian carcinoma cells. Induction of AKT activation causes tumorigenesis and becomes a problem for treating cancer [26, 27]. It must be treated together inhibiting of AKT activity as well as increasing apoptosis [28, 29]. Recent studies showed that RVS reduces AKT phosphorylation in gastric cancer cell lines [30] and that n-butanol extract form RVS decreases LPS-induced AKT activation in macrophage RAW 264.7 cells [31]. Therefore, we evaluated the effects of RVS in paclitaxel-resistant ovarian cancer cell lines.

The results indicate that RVS was lead to apoptosis via both accumulation of cleaved PARP form and suppression of AKT activation in a dose-dependent manner. Furthermore, we conducted experiments to know what compounds of RVS have these capabilities. Among the three compounds, butein effected best, reducing the proliferation of SKOV-3/PAX cells. Butein, a bioactive flavonoid [32, 33], was effective for cancer treatment through apoptosis pathway such as caspases and PARP. Also, Butein affected the cell proliferation via repressing AKT phosphorylation in cervical cancer cells and AKT-dependent phosphorylation of hTERT in leukemia cells [32, 34]. It is reported that other compounds, fisetin and quercetin, induce apoptosis in cancer cells, but slightly inhibited proliferation in SKOV-3/PAX cells [24, 25]. Lastly, after treatment of SKOV-3/PAX cells with PI3K inhibitor LY294002, the function of AKT was repressed. The result is RVS and butein can induce SKOV-3/PAX cell apoptosis by reducing AKT pathway activation. Accordingly, the present study suggested that the inhibiting AKT phosphorylation is a key role for the apoptosis of paclitaxel-resistant ovarian cancer cells.

## Conclusion

We show that RVS and its active compound, butein, may enhance apoptosis and reduce proliferation through the inhibition of AKT activation in paclitaxel resistant-ovarian cancer. Therefore, our results suggested that RVS could be useful for the chemotherapy of drug-resistant cancer and more researches are needed to further a detailed mechanism.

### Ethics

This study used cell lines commercially available, thereby not requiring the ethics.

### Availability of data and materials

Data are all contained within the paper.
